# Online Training That Changes Family Caregiver Behavior and Attitudes

**DOI:** 10.1093/geroni/igab046.1883

**Published:** 2021-12-17

**Authors:** Vicki de Klerk-Rubin

**Affiliations:** Validation Training Institute, Pleasant Hill, Oregon, United States

## Abstract

The Family Caregiver Course (FCC) is an 18-week Validation training to sensitize family carers to the psycho-social needs of their relatives, integrate new behaviors that build relationships and specific verbal and non-verbal techniques to increase communication. Validation Training Institute partnered with the Alzheimer’s Association of Colorado in 2019 and 2020 in delivering this course. Due to COVID-19 limitations, the 2020 iteration was completely digital, using principles of online learning. To replace the two-day in-person component of this course, we developed four, 4-hour Zoom sessions that allowed participants to: practice specific Validation techniques, exercise, process and apply what was learned, role play and receive coaching to anchor skills. Important online rules were maintained, such as, offer opportunities for participant engagement every 10 minutes and create a community of inquiry. Pre- and Post-Surveys of the 2019 and 2020 iterations of FCC showed that after taking this course, family carers: reported that they knew what to do when faced with challenging behaviors from their relative; understood that lying or pretending to agree with a disoriented person was not an effective strategy for communication; gained knowledge of the different forms of dementia and that the differences are significant; were clear about the differences between Validation and other methods.

